# Insights into the Role of Galectin-3 as a Diagnostic and Prognostic Biomarker of Atrial Fibrillation

**DOI:** 10.1155/2023/2097012

**Published:** 2023-10-09

**Authors:** Yasmin Mohtasham Kia, Alessandro Cannavo, Pegah Bahiraie, Sanam Alilou, Behrad Saeedian, Nastaran Babajani, Elina Ghondaghsaz, Amirmohammad Khalaji, Amir Hossein Behnoush

**Affiliations:** ^1^School of Medicine, Iran University of Medical Sciences, Tehran, Iran; ^2^Department of Translational Medical Sciences, Federico II University of Naples, Naples, Italy; ^3^School of Medicine, Shahid Beheshti University of Medical Sciences, Tehran, Iran; ^4^School of Medicine, Tehran University of Medical Sciences, Poursina St., Keshavarz Blvd., Tehran 1417613151, Iran; ^5^Undergraduate Program in Neuroscience, University of British Columbia, Vancouver, BC, Canada

## Abstract

Atrial fibrillation (AF) is an irregular atrial activity and the most prevalent type of arrhythmia. Although AF is easily diagnosed with an electrocardiogram, there is a keen interest in identifying an easy-to-dose biomarker that can predict the prognosis of AF and its recurrence. Galectin-3 (Gal-3) is a beta-galactoside binding protein from the lectin family with pro-fibrotic and -inflammatory effects and a pivotal role in a variety of biological processes, cell proliferation, and differentiation; therefore, it is implicated in the pathogenesis of many cardiovascular (e.g., heart failure (HF)) and noncardiovascular diseases. However, its specificity and sensitivity as a potential marker in AF patients remain debated and controversial. This article comprehensively reviewed the evidence regarding the interplay between Gal-3 and patients with AF. Clinical implications of measuring Gal-3 in AF patients for diagnosis and prognosis are mentioned. Moreover, the role of Gal-3 as a potential biomarker for the management of AF recurrence is investigated. The association of Gal-3 and AF in special populations (coronary artery disease, HF, metabolic syndrome, chronic kidney disease, and diabetes mellitus) has been explored in this review. Overall, although further studies are needed to enlighten the role of Gal-3 in the diagnosis and treatment of AF, our study demonstrated the high potential of this molecule to be used and focused on by researchers and clinicians.

## 1. Introduction

More than a century ago, atrial fibrillation (AF) was first discovered [1], and this disorder has been acknowledged as the most common type of cardiac arrhythmia, primarily found in the elderly population, and that has increased the rates of morbidity, disability, and mortality with substantial healthcare costs. Recent reports estimated that more than 30 million people worldwide will have AF by 2050 [2], demanding scientists' urgent commitment to finding a way to arrest this epidemic disease.

During the last decades, clinical classification of AF has been used to communicate the persistence of the disease, individualize the choice of rate or rhythm control procedures, and select proper medical or interventional treatments for each patient [3–5]. Indeed, AF is typically subdivided into paroxysmal if arrhythmic episodes last less than 7 days and cease spontaneously or by cardioversion [6]. Alternatively, when arrhythmia is sustained for more than 7 days and/or demands intervention to terminate, AF is then classified as persistent and nonparoxysmal. If pharmacological or electrical cardioversion fails or has not been endeavored, AF is conventionally defined as permanent AF.

An accurate diagnosis of AF is essential as this disorder is considered an independent risk factor for several chronic disorders, like heart failure (HF), dementia, and cognitive dysfunction, which represent a major cause of increased morbidity and mortality, especially in the aged population [7–11]. Moreover, irregular beatings can be responsible for blood clot formation, resulting in a five-fold increase in stroke risk and a two-fold increase in death rate compared to other disorders [12–14]. Unfortunately, there is high uncertainty in diagnosing AF [3], especially for paroxysmal AF, because there may be no manifestation of the disease early in these patients, and there is a spontaneous cessation of arrhythmia [15]. In addition, a third of patients are asymptomatic (silent AF) and escape recognition until AF is detected on a regular medical visit or when its comorbidities that demand urgent medical attention develop, significantly impacting the real burden of this disorder [16]. In these patients, therapy is not initiated and is likely to result in adverse patient outcomes [17, 18].

Although several risk factors have been identified so far, and the scoring systems applied for AF, it is still a challenging impediment to probing and elucidating the etiology of AF and seeking complementary therapeutic approaches and strategies to reduce the burden of this disease. Also, there is a trend toward finding relevant biomarkers for AF, and thus, there is a high interest in identifying specific biomarkers that can aid in risk stratification among patients with AF for different causes of death.

In this review, we focused on Galectin-3 (Gal-3) as a *β*-galactoside-binding protein with a pivotal role in a broad range of biological processes, controlling cell proliferation, differentiation, and survival, and considered a promising biomarker of inflammatory and fibrotic-based disorders including HF. Although the association between Gal-3 and HF is well established [19–21], the potential application of Gal-3 in the prognosis and diagnosis of AF is still under investigation as it demands further molecular basis and clinical studies to understand the role of this Gal-member fully. However, although controversial and highly debated, the prognostic role of Gal-3 in AF is more than plausible [22].

Indeed, AF is strictly accompanied by a structural remodeling of the atrial myocardium with consistent pro-inflammatory and -fibrotic changes that lead to conduction abnormalities [23, 24], effects associated with Gal-3 activity. To date, despite being investigated in individual studies, there has been no comprehensive review of the interplay between AF and Gal-3. In keeping with this view, this review will discuss the most important and novel clinical and experimental evidence around the usefulness of Gal-3 as a diagnostic and prognostic biomarker in AF.

## 2. Biology of Gal-3: Structure and Functions

Galectins (Gals) are a family of evolutionarily conserved proteins containing either a single or two conserved carbohydrate recognition domains (CRDs) of about 135 amino acids that confer to these proteins a high affinity for *β*-galactosides [25].

There are 15 reported components of the Gal family in mammalians, and based on CRD, these proteins can be classified into three groups [26, 27]:

1. Prototype Gals with a single-CRD include Gal-1, -2, -5, -7, -10, -11, -13, -14, and -15.
2. Tandem-repeats Gals with two CRDs include Gal-4, -6, -8, -9, and -12.
3. The chimera-type Gals with a CRD at the C-terminus (CT) linked to an N-terminus (NT) collagen-like domain participating in the oligomerization of Gal molecules and the interactions with different proteins [27–29].

**Figure 1:**
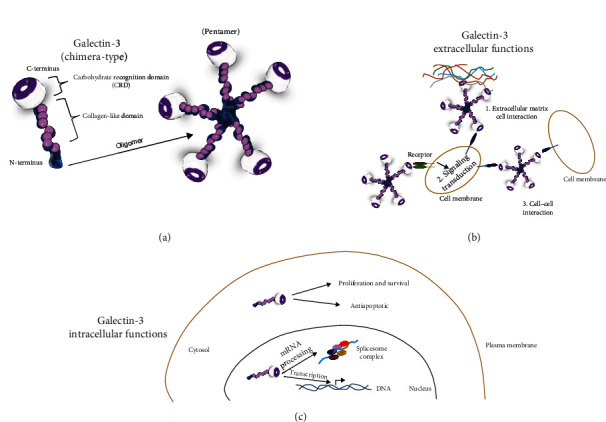
Schematic representation of structure and functions of galectin-3. (a) Galectin-3 belongs to the chimera-type group presenting carbohydrate recognition domains (CRDs) at the C-terminus (CT) linked to an N-terminus collagen-like domain that participates in the formation of oligomers like the pentamer of galectin-3. (b) Extracellular and (c) intracellular (cytosolic and nuclear) functions of Galectin-3.

Among the Gals members, Gal-3 is the only one belonging to the chimera group, and its structure is characterized by an NT that is crucially involved in the regulation of intracellular signaling and the formation of oligomers and a CRD that interacts with glycoproteins, including cytokine receptors and growth factors [30]. Like the other Gal members, Gal-3 is a soluble protein with no transmembrane domain and elicits several activities in nuclear and cytoplasmic compartments. Moreover, Gal-3 can also be secreted into the extracellular space and circulation [31, 32], becoming a multifaceted functional protein with an essential role in lots of physiological and pathological processes (Figure 1). For instance, Gal-3 has been shown to exert antiapoptotic effects and control cell proliferation when confined to the cytoplasm [25]. While in the nuclei, Gal-3 is involved in gene transcription regulation and mRNA splicing mechanism. These effects are strictly regulated also via a complex interactome that includes proteins such as *β*-catenin, CBP70, RAS proteins, Gemin4, Chrp, Alix/AIP-1, Bcl-2, and factors of the nuclear spliceosome complex [25]. In the alternative, when present on the cell surface or secreted in the extracellular environment, Gal-3 is involved in multiple processes, including membrane receptors signaling transduction, cell adhesion and migration, growth regulation, and cell-death control (pro-apoptotic effects). Despite these multiple activities, extensive experimental pieces of evidence implicate Gal-3 in tissue inflammation and fibrosis [32, 33]. Indeed, this protein is mainly expressed by macrophages, which regulate alternative macrophage activation (M2) and neutrophils, into which Gal-3 acts as an inhibitor of apoptosis, thereby leading to increased infiltration, inflammation, and tissue fibrosis. Moreover, Gal-3 is a well-recognized enhancer of collagen production/deposition and secretion of interleukin (IL)-1, IL-6, and monocyte chemoattractant protein-1 from fibroblasts and acts as a trigger stimulus for the transformation of quiescent fibroblasts into myofibroblasts [34].

## 3. Gal-3 and AF

Based on multiple clinical and preclinical studies demonstrating an association between altered peripheral Gal-3 levels and interstitial fibrosis and inflammation, with consequent organ (mainly heart and lung) failure [35–37], in the 2017 guidelines, the American Heart Association recommended Gal-3 measurement for the prognosis of HF [38], and circulating Gal-3 levels were suggested as biomarkers of cardiovascular diseases (CVDs) and non-CVDs [39–41]. Moreover, recent studies have proposed serum Gal-3 levels as strictly associated with atrial remodeling, including the extent of atrial fibrosis [42]. However, as Gal-3 is a potent inflammatory protein with the contribution to the initiation and amplification of the inflammatory response, it increases in other diseases than CVDs, including kidney [43], liver [44, 45], pulmonary diseases, apnea [46, 47], and also infectious diseases [48]. This might confine the specificity of this biomarker despite the fact that all CVDs associated with an increase in Gal-3 have some sort of fibrosis or remodeling in their pathogenesis [49]. Moreover, due to the pleiotropic actions of Gal-3, possible roles of this biomarker might be observed in cardiomyocytes, such as modulation of cardiac ion channels. This includes the interaction of Gal-3 with beta-galactoside residues of cell surface and matrix glycoproteins [50]. Also, based on evidence, the interaction of galectin–N-glycan in the kidney can prevent the internalization of ROMK1 and transient receptor potential cation channels [51]. Similarly, Gal-3 might prevent the internalization of K-ion channels like Kir2.3 and Kv1.5 [52–54]. Oligomers of Gal-3 can bind to these two ion channels in atrial myocytes, leading to the shortening of action potential and consequently AF progression [55].

One of the first studies reporting a role for Gal-3 in AF was the report by Sonmez et al. [56]. These authors observed significantly higher serum Gal-3 levels in patients with AF compared to those with sinus rhythm. Further, they demonstrated that Gal-3 peripheral levels can serve as a predictor of the left atrium (LA) remodeling onset. In line with these data, Gurses et al. [57] reported significantly higher serum Gal-3 in AF patients compared with the control group. Moreover, these authors observed that serum Gal-3 levels were also significantly higher in patients with persistent AF compared to patients with paroxysmal AF (*p*-value < 0.001), results further confirmed by Va et al. [58] and a meta-analysis conducted by Gong et al. [23].

Next, the study by Yalcin et al. [59] measured serum levels of Gal-3 in patients with paroxysmal AF with preserved left ventricular (LV) functions, demonstrating that this factor correlated with the extent of LA fibrosis, detected by delayed enhancement magnetic resonance imaging. Similar results were also obtained by Hernández-Romero et al. [60] and showed that high Gal-3 serum levels could predict atrial appendage fibrosis in permanent AF compared to their counterparts.

In another study, Chen et al. [61] demonstrated that new-onset AF was associated with elevated Gal-3 levels comparable to those with preexisting, chronic AF, supporting the potential role of Gal-3 as a biomarker of AF chronicity. In line with this idea, Wang et al. [62] analyzed the levels of plasma Gal-3 in a cohort of 51 patients with paroxysmal AF progressed to persistent AF. The authors demonstrated that the elevated Gal-3 level as a biomarker was significantly associated with AF progression, confirming the potential role of Gal-3 in patient stratification. Selcoki et al. [63] reported that serum Gal-3 levels are significantly elevated and correlated with LA diameter (LAD) in patients with paroxysmal AF.

In the Atherosclerosis Risk in Communities study, Fashanu et al. [64] evaluated the association of Gal-3 with AF incidence in a cohort of about 8,000 individuals, showing that elevated levels of Gal-3 were associated with an increase of more than 40% in AF incidence in the general population. A recent systematic review of 12 studies also evaluated the association between Gal-3 peripheral levels and AF, of which some were mentioned before [65]. They found that peripheral Gal-3 levels are higher in nonvalvular AF patients, whether in a persistent or paroxysmal form and that it can be used as a marker for fibrosis. Another recent meta-analysis with more than 10,000 patients [23] came to four important conclusions: first, patients with AF had higher levels of Gal-3 compared with the sinus rhythm group (mean difference: −0.68 ng/mL, 95% confidence interval (CI): −0.92 to −0.44, *p*-value <0.00001). Second, higher Gal-3 levels increased the likelihood of developing AF by 45% (odds ratio (OR) 1.45, 95% CI 1.15–1.83, *p*-value = 0.002). Third, six of those 28 studies compared Gal-3 circulating levels between patients with paroxysmal and persistent AF and showed that it is significantly higher in patients with persistent AF. Finally, patients with no recurrence after treatment had substantially lower levels of Gal-3 compared to patients with AF recurrence.

There have also been recent studies regarding Gal-3 and AF, such as one by Pauklin et al. [66], which measured several biomarkers of oxidative stress, fibrosis, and inflammation in patients with AF to define their role in this disease. The results were consistent with previous studies and found that Gal-3 is significantly higher in patients with AF compared with healthy subjects (11.4 vs. 9.7 mg/L, *p*-value = 0.003). Lastly, a recent study analyzed the relationship between levels of Gal-3 genotyping and persistent AF. They found that between the three polymorphisms in patients (Rs2274273, Rs1558648, and rs13019803), Rs2274273 was significantly related to the levels of Gal-3 [67]. This field might improve our understanding of Gal-3 and its role in developing AF and needs further research and exploration.

Importantly, human studies usually result in heterogeneous results. Indeed, while some investigations (as discussed above) showed that circulating Gal-3 levels are higher in patients with AF and are correlated with the degree of atrial fibrosis, other studies have found no considerable differences [68]. A prospective study with more than 3,000 subjects (Framingham Offspring cohort) found that high Gal-3 levels predicted the incidence of AF in the general community [69]. However, when accounted for other risk factors, Gal-3 failed to predict AF risk.

Finally, it should be noted that AF might be present in patients with other arrhythmias. There have been limited reports on the association of Gal-3 and these arrhythmias as well. For example, it has been suggested that patients with ventricular arrhythmia storm had significantly higher levels of Gal-3 compared to those without shocks, while Gal-3 had 84% sensitivity and 75% specificity for indication of ventricular arrhythmias requiring therapies [70]. In the study by Akbulut et al. [71], it was shown that patients with higher levels of Gal-3 experienced higher rates of nonsustained ventricular tachycardia, sustained ventricular tachycardia, and ventricular fibrillation, while it was suggested a predictor of ventricular arrhythmias in patients with HF.

## 4. Management of AF: Gal-3 and AF Recurrence

Recurrence of AF is common, with ranges from 40% to 70%, despite the administration of antiarrhythmic drugs and the attempts of cardioversion [72–74]. This proportion can be reduced to 45% up to 20% after catheter ablation [75, 76], an effective treatment in patients with symptomatic AF [77] that allows for achieving a better quality of life than other approaches [78, 79]. In addition, AF ablation may positively affect LV function in patients with HF [80]. Biomarkers have been suggested in some instances of AF, e.g., in patients with metabolic syndrome having AF, for which Gal-3 and growth/differentiation factor-15 (GDF-15) were suggested to be predictors of AF recurrence [81]. This can have implications for personalizing antiarrhythmic medications.

Ablation is the electrical isolation of pulmonary veins and is associated with defragmentation. Different outcomes, for instance, a higher rate of recurrence, can happen after ablation in patients with persistent AF and depends on the procedure method and clinical presentation of the disease [82]. Identifying potential predictors of success in keeping sinus rhythm after ablation or cardioversion may permit a more suitable selection of patients to undergo these therapeutic approaches and could have an even more significant impact on the recurrence of AF.

For instance, as atrial fibrosis is known to be a major responsible factor for arrhythmogenic effects, it is considered a predictive factor of AF recurrence after ablation [83]. Therefore, a quantitative approach to estimating the amount of fibrosis may be a helpful indicator of ablation outcomes [83]. In this regard, Begg et al. [83] used cardiac magnetic resonance imaging (CMR) to detect the size of fibrotic tissue in the LV. These authors measured LA fibrosis using invasive LA voltage mapping and LA pressure, finding an association between these parameters and AF recurrence. Similarly, the DECAAF study highlighted the role of atrial fibrosis patients who underwent AF ablation [84]. However, CMR, the method used to quantify atrial fibrosis, appears cost-ineffective compared to a simple dosage of Gal-3 serum level. Moreover, Gal-3 levels in AF patients can be related to several AF-related disorders, including obesity, hypertension, or diabetes (metabolic syndrome), that are known to facilitate favor fibrogenesis [85]. In this scenario, Kornej et al. [24] evaluated the relationship between the baseline Gal-3 levels in AF patients and the non-AF cohort with comorbidities. These authors concluded that cardiometabolic comorbidities and not heart rhythms mostly drove the observed Gal-3 levels in AF patients.

In another prospective cohort study by Lee et al. [86], nonparoxysmal AF (hazard ratio (HR) 6.8; 95% CI 1.6–28.9) and higher Gal-3 levels (HR 1.3; 95% CI 1.0–1.7) were associated with an increased risk of recurrence. Moreover, serum Gal-3 levels were more elevated in patients with diabetes and were associated with values that denoted the LA size. Next, Clementy et al. [87] assessed the potential value of this biomarker in monitoring AF recurrence after ablation. Notably, this study showed that Gal-3 levels are a strong predictor of AF recurrence, independently of whether the type of AF is paroxysmal or persistent. Moreover, these authors suggested that if Gal-3 levels are combined with LAD measurement, they can identify patients at different risks of arrhythmia recurrence (low, intermediate, and high) at 1 year. In line with this study, Wu et al. [88] reported that both Gal-3 (HR 1.28, *p*-value = 0.006) and LAD (HR 1.1, *p*-value = 0.025) were independent predictors of AF recurrence after ablation. In addition, adding Gal-3 levels to LAD had an additive predictive value for outcomes following AF ablation [88]. However, several other studies count the Gal-3 value as a promising independent predictor of AF recurrence [55].

Systematic reviews and meta-analyses were conducted to clarify better the predictive role of Gal-3 levels for recurrence after catheter ablation [89, 90]. Zhang et al. [89] found that a 1 ng/mL increase in plasma Gal-3 levels was associated with about a 17% increase in the risk of AF recurrence, independent of age, gender, and baseline LA dimension. Similar to these findings, Paranata et al. found that serum Gal-3 was associated with an increased risk of AF recurrence after cardiac ablation. Pooled analysis of adjusted HRs established that high serum Gal-3 is an independent predictor of AF recurrence (HR 1.15) [90].

However, other studies found the opposite results. For instance, Lopez et al. [91] indicated that Gal-3 provided no added predictive value for AF recurrence. Similarly, Begg et al. [92] found that Gal-3 does not predict AF recurrence postablation. LA voltage independently predicted AF recurrence, whether the LA was mapped in AF or sinus rhythm [92]. Berger et al. [93] also failed to find an association between peripheral Gal-3 levels and AF recurrence after thoracoscopic ablation. Contrarily, they found that changes in Gal-3 values were predictive. Further, Celik et al. [94] found no association between Gal-3 levels and AF recurrence at 6 and 12 months after ablation. However, Gal-3 levels were raised or reduced in the AF recurrence and nonrecurrence groups, respectively, even though these changes were not statistically significant.

Other studies evaluated the correlation between Gal-3 and elective direct current cardioversion (DCCV). In this regard, Walek et al. [95] demonstrated that circulating Gal-3 levels had a significant value in diagnosing severe grade LV diastolic dysfunction after successful elective DCCV. These authors observed that a rise in concentrations of Gal-3 negatively correlated with the volume and contractility of the LV, with the LA size and fibrosis, systolic function, and compliance of the LA wall in those patients with persistent AF. In another study by Walek et al. [96], the levels of Gal-3 were prognostic in terms of maintaining sinus rhythm after DCCV. Finally, Gurses et al. [97] showed that the Gal-3 levels were augmented in patients with persistent AF than in patients with paroxysmal AF following successful DCCV. They also showed a significant positive correlation between Gal-3 levels and with LA volume index. In contrast with these studies, Merino-Merino et al. [98] found that Gal-3 concentrations, measured at DCCV and 6 months post-DCCV, were not prognostic for maintaining sinus rhythm.

## 5. Gal-3 as a Potential Biomarker of AF in Special Populations

**Figure 2:**
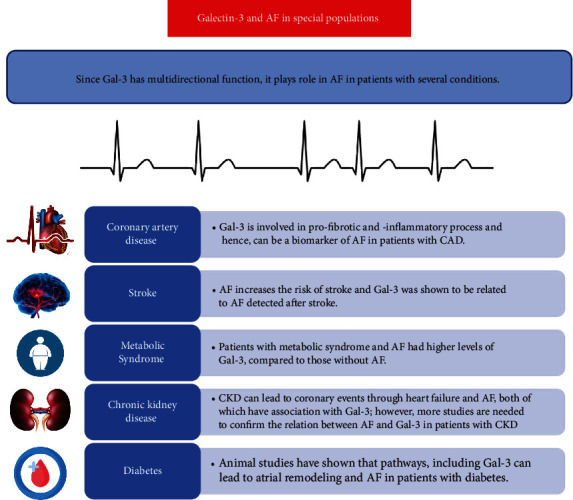
Summary of studies investigating the association of Gal-3 and AF in special populations. AF, atrial fibrillation; CAD, coronary artery disease; CKD, chronic kidney disease.

Due to its multidirectional function, Gal-3 plays a significant role in several clinical statuses and disease natures [26]. The Gal-3 presentation has been documented in a variety of cardiac and noncardiac conditions in companies with AF, such as coronary artery and other heart diseases, metabolic syndrome, diabetes, chronic renal failure, as well as ischemic stroke. A summary of the findings of studies for the association of Gal-3 and AF is shown in Figure 2.

### 5.1. Coronary Artery Disease (CAD)

The entity of CAD includes necrosis of the myocardium, infarcted tissue inflammation, and fibrosis. AF is associated with the atria's contractile, structural, and electrical remodeling [99], and Gal-3 is likely involved in CAD and AF progress, being involved in the pro-fibrotic and -inflammatory processes [100]. Importantly, Kang et al. [100] showed that the plasma concentration of Gal-3 augmented remarkably with the severity of the myocardial ischemia. These authors also found that the levels of this biomarker were significantly higher in patients with AF compared to healthy individuals. Further, when AF patients underwent radiofrequency ablation, Gal-3 serum concentration decreased [100]. Studies have investigated Gal-3 concentrations in patients with AF rhythm and CAD [101–107], a summary of which is represented in Table 1.

#### 5.1.1. Post-Myocardial Infarction (MI)

New-onset AF is the most prevalent tachyarrhythmia after acute MI (AMI) [109]. Numerous investigations compared the entities of AMI patients with and without new-onset AF during hospitalization courses and surveyed Gal-3 plasma levels. A retrospective cohort found that in hospitalized patients with acute MI, baseline plasma Gal-3 levels augmented in patients with new-onset AF, supporting the potential value of Gal-3 as an independent predictor of AF [101]. In a randomized controlled trial, Kalstad et al. [103] studied patients aged 70–82 with MI. Among the biomarkers tested, the sirtuin-1 and the soluble form of the suppression of tumorigenicity 2 factor, Gal-3, was the only one not associated with AF. Conversely, according to Pavlović et al. [102] study, serum Gal-3 level, above the defined threshold, was the only marker predicting AF. However, in most studies [102, 103], Gal-3 plasma levels in patients with AMI and AF were higher than those with MI without AF. Finally, Stanojevic et al. [104] observed that Gal-3 levels were significantly higher in permanent/persistent and paroxysmal AF compared to acute MI patients without AF.

#### 5.1.2. Post-Cardiac Surgery

Postoperative AF during the first days after cardiac surgery occurs in about 20%–50% of patients and is strictly associated with thromboembolic events, instability in hemodynamic conditions, HF, longer hospitalization, and damaging outcomes [110]. Intuitively, Gal-3 can provide a discriminatory prospect of postsurgery AF. Although Gal-3 level may be a lightly measurable indicator of postoperative AF and fibrosis of the atrium, it does not only represent fibrosis in atrial tissue but also reflects fibrosis in the chamber of ventricles, which causes HF and in other organs like the kidney, liver, and lung [106]. Contrary to the specificity absent for atrial tissue, Gal-3 might be a good predictor of AF incidence after cardiac surgery, including coronary artery bypass graft (CABG).

A study by Alexandre et al. [105] in patients undergoing elective CABG found significantly higher levels of Gal-3 in patients with postoperative AF compared to controls. In line with these results, Richter et al. [106] found preoperative Gal-3 levels a predictor of postoperative AF, higher in patients with postoperative AF than those without AF. Finally, in a retrospective cohort, Gal-3 and Growth/differentiation factor 15 showed promising results as a biomarker of chronic AF in advanced CAD candidates for cardiac surgery [107].

#### 5.1.3. HF

AF is the most common arrhythmia in HF and is linked with several circulating prognostic and diagnostic biomarkers of HF [111]. However, there is a knowledge gap about whether markers representing fibrosis in heart tissue are related to HF and simultaneous AF patients' prognosis. Applying fibrotic myocardial biomarkers in clinical practice entails an outstanding knowledge of the two-sided pathophysiology between HF and AF [112]. In this context, Merino-Merino et al. [113] analyzed the importance of some biomarkers, including NT-proBNP, high-sensitivity troponin T (hsTnT), ST2, C-reactive protein, fibrinogen, urate, and Gal-3 in diagnosing patients with symptomatic and persistent AF with or without HF with preserved LV ejection fraction (HFpEF). Further, a difference between medium-range ejection fraction HF (HFmrEF) and those with HFpEF was also analyzed. These authors found that the only indicator that yielded statistical significance was NT-pro-BNP, while Gal-3 and hsTnT were approximately significantly related. However, TnT was the only factor independently associated with HFmrEF. In another study, Nezami et al. [112] evaluated plasma levels of several proteins formerly correlated with myocardial fibrosis (metalloproteinase inhibitor 4 (TIMP-4), ST-2, Gal-3, GDF-15, and matrix metalloproteinase 2, 3, and 9) in hospitalized patients with HF. Of note, these authors found that increased concentrations of five plasma proteins, including Gal-3, were significantly associated with all-cause mortality in patients with coexisting AF. For their part, Tan et al. [114] investigated the impact of AF on the prognostic performance of different biomarkers in HF. The primary outcome of their study included mortality rate or hospital admission. Among the biomarkers tested, Gal-3 predicted HF hospitalization only in patients with AF [114].

One of the most important points that need to be mentioned is atrial dysfunction and AF secondary to arrhythmogenic ventricular remodeling since atrial dysfunction can result from ventricular remodeling. In this regard, it has been shown that serum Gal-3 was increased in patients with arrhythmogenic right ventricular dysplasia with the potential to become a diagnostic biomarker [115]. Further studies should assess the role of Gal-3 in patients with arrhythmogenic ventricular remodeling.

### 5.2. Stroke

As discussed above, AF increases the risk of stroke and is related to more than 1/5 of ischemic stroke cases [12]. A novel prospective study by Garnier et al. [116] studied the pathophysiology of AF detected after stroke (AFDAS) in 1,796 patients through a multimodal method that consisted of radiological, biological, electrocardiogram, and clinical markers such as LA size and function, serum level of fibrosis biomarkers, cardiac remodeling, and inflammatory mediators. Based on this model, these authors observed that Gal-3 (≥9 ng/mL) was independently related to AFDAS as its serum levels were higher in AF patients and were associated with LA volume, foreshowing AF incidence and postablation recurrence.

### 5.3. Metabolic Syndrome

Epidemiological knowledge expresses the wide spreading of AF in the young population with no common risk factors for this kind of tachyarrhythmia (e.g., CAD, HF, and valvular diseases) but with central obesity and high blood pressure, which are independent predisposing causes for AF [117]. Hypertension and high body mass index are the most current incorporators of metabolic syndrome, which can motivate conversion in the atrial structure, such as increased dimension and fibrosis of the LA [118]. Ionin et al. [119] studied about 1,300 hospitalized patients to determine the blood concentration of Gal-3 and establish whether this factor was associated with procollagens and myocardial fibrosis in metabolic syndrome cases from 2014 to 2018. Notably, the results of this study demonstrated that the plasma levels of Gal-3 were significantly higher in patients with both AF and metabolic syndrome than in those without AF and in the control group.

### 5.4. Chronic Kidney Disease (CKD)

HF and AF are the foremost causes of coronary events in patients with CKD and are related to more significant mortality and other adverse outcomes [120]. Multiple hypotheses can explain the relationship between CKD and myocardial structure and conduction modifications. Different blood biomarkers are presumed to be associated with these changes [121], and recent studies have examined the relationship between Gal-3 and AF occurrence in CKD. However, most of these studies represented a common opinion that this association was not seen in schematic analyses and that Gal-3 levels were not linked to the AF risk [120–122].

### 5.5. Diabetes

Diabetes is an independent cause of AF and has been assumed to be associated with myocardium remodeling, as the increased inflammatory reactions, typical of diabetes, may be an effective mechanism of atrial changes [123]. Although the literature about serum biomarkers and their association with AF in diabetic patients is limited, an animal study (diabetic rabbits) by Wu et al. [124] established that increased LRP3-inflammasome/caspase-1/Gal-3 pathway activity impacts atrial remodeling and is therefore implicated in AF induced by diabetes.

## 6. Aldosterone and Gal-3 in AF

Like Gal-3, aldosterone is a well-recognized cardiac fibrosis and inflammation marker and is implicated in many CVDs [125]. In addition, several experimental and clinical studies underlined the direct interaction of aldosterone with Gal-3 and the association of these pathways with cardiac fibrosis and adverse remodeling [36, 126]. Indeed, aldosterone facilitates Gal-3 expression and secretion, negatively affecting disparate tissues and organs systemically.

In this context, a study by Liao et al. [127] demonstrated that patients with aldosterone-producing adenomas had increased myocardial fibrosis strictly associated with higher plasma Gal-3 levels. Of note, these authors also showed that myocardial fibrosis and Gal-3 levels decreased after adrenalectomy, thus supporting the relationship between aldosterone and Gal-3. Considering this observation, our group studied the potential biomarker role of Gal-3 and aldosterone in predicting cardiovascular complications in COVID-19 patients [33].

Concerning AF, several studies investigated the implication of aldosterone, as this hormone has been shown to cause atrial fibrosis independently of changes in wall stress or hypertension. At the same time, its blockade via spironolactone (aldosterone-receptor, mineralocorticoid receptor (MR) antagonist) prevents these effects [128]. Analogously, eplerenone a more selective MR antagonist [129] has been shown to suppress inducible AF in experimental HF [130]. Human studies have demonstrated that plasma aldosterone levels significantly increase in AF patients [131] and MR expression rises in the atria of these patients [132]. Unfortunately, the studies correlating Gal-3 and aldosterone are limited. Alexandre et al. [105] demonstrated that patients experiencing postoperative AF following CABG had higher preoperative plasma aldosterone levels than controls and that this hormone was independently associated with postoperative AF. Moreover, these authors revealed that Gal-3 was similarly activated in postoperative AF patients in line with the aldosterone trend. Further, Ruan et al. [133] investigated whether Gal-3 levels were predictors of AF recurrence after radiofrequency catheter ablation. These authors observed that AF recurrence after radiofrequency catheter ablation presented with higher baseline Gal-3 and aldosterone levels. Further, they demonstrated that more elevated preoperative levels of these factors can independently predict AF recurrence in these patients.

## 7. Conclusions

**Figure 3:**
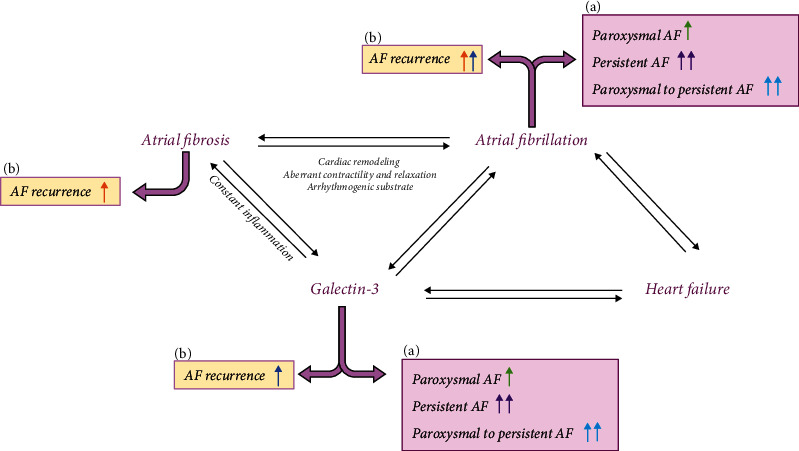
The association between galectin-3, atrial fibrillation, and heart failure in recurrence and prognosis of AF (matching colors show associations). (a) Higher Gal-3 is associated with paroxysmal AF, persistent AF, and paroxysmal-to-persistent AF. (b) Higher levels of Gal-3 in AF recurrence based on preclinical studies.

Overall, this review article has explored the potential role of Gal-3 as a biomarker of AF. Moreover, we have investigated its role as an AF recurrence predictor, an event expected in the patients affected by this disorder that can lead to or participate in the development/progression of other diseases, including stroke. Gal-3 is a pro-fibrotic and -inflammatory molecule highly implicated in the pathogenesis of different systemic disorders, including AF. In this regard, Figure 3 illustrates key concepts regarding the role of Gal-3 and interactions between AF, atrial fibrosis, HF, and AF recurrence. Based on this premise, studies support Gal-3 inhibition as a therapeutic approach to fight AF and enhance outcomes of AF therapies like catheter ablation [55].

Herein, we have provided an update about the clinical studies in which this biomarker role has been suggested. Interestingly, despite the assessment of Gal-3 levels in AF patients sometimes appearing controversial, the general idea is that this easy-to-dose molecule may be a valuable biomarker of AF progression and recurrence. Of course, future research focusing on this novel biomarker is warranted to better understand its clinical use and possible therapeutic capabilities. Analogously, it will be essential to investigate the interrelation between Gal-3 and other pro-fibrotic molecules acting on the same signaling pathways, like aldosterone or the transforming growth factor-*β*. The latter was recently investigated by Takemoto et al. [55] in an animal model (sheep) of AF, demonstrating how Gal-3 via TGF-*β* causes atrial fibrosis.

## Figures and Tables

**Table 1:** Gal-3 in patients with AF and CAD.

Study	Study design	CAD	Population	Study group	Control group	Sample size	Age	Main findings
Wang et al. [108]	Retrospective cohort	MI	Hospitalized patients with AMI	NOAF (*N* = 18)	Sinus rhythm (*N* = 199)	217	58.3 ± 10.7	Plasma levels of Gal-3 were significantly increased in AMI patients with NOAF and is an independent predictor of NOAF

Pavlović et al. [102]	Prospective case–control	MI	Non-ST elevation acute MI	Preexisting AF (*N* = 22)	Sinus rhythm (*N* = 32)	54	68.1 ± 11.0	Gal-3 plasma levels in patients with the first acute NSTEMI were significantly higher in preexisting AF patients compared to non-AF patients. Gal-3 levels also have shown a significant and independent predictive value of recurrence after RF ablation for AF

Kalstad et al. [103]	Retrospective cohort	AMI	Hospitalized elderly patients with AMI	Preexisting AF (*N* = 38)	Sinus rhythm (*N* = 261)	299	75 (72–78)	They found no association between Gal-3 levels and the presence of AF in AMI patients

Stanojevic et al. [104]	Retrospective cohort	AMI	Patients with first AMI without revascularization history	Permanent/persistent AF (*N* = 27) and paroxysmal AF (*N* = 24)	Sinus rhythm (*N* = 38)	89	66.3 ± 11.3	Gal-3 and hs-CRP levels were significantly higher in AMI patients with AF compared to those without permanent/persistent/paroxysmal AF

Alexandre et al. [105]	Prospective cohort	Cardiac surgery	LVEF > 50% requiring elective CABG	POAF (*N* = 34)	Sinus rhythm (*N* = 103)	137	67.2 ± 10.7	Gal-3 levels were significantly higher in the POAF group compared to sinus rhythm (14.5 ng/mL (9.2–15.6) vs. 7.4 ng/mL (5.5–8.3); *p*-value = 0.002)

Richter et al. [106]	Prospective cohort	Cardiac surgery	Elective heart surgery	POAF (*N* = 200)	Sinus rhythm (*N* = 275)	475	67.4 ± 11.8	Preoperative Gal-3 levels were significantly higher in the POAF group compared to patients without POAF (9.60 ± 6.83 ng/mL vs. 7.10 ± 3.54 ng/mL; *p*-value < 0.001)

Doulamis et al. [107]	Retrospective cohort	Cardiac surgery	Advanced CAD candidates for cardiac surgery	Chronic AF (*N* = 22)	Sinus rhythm (*N* = 101) and healthy controls (*N* = 20)	143	64.2 ± 11.9	An optimal pair of cytokines, including Gal-3 and others (e.g., CXL-10, CXCL-10, GDF-15, and RETN), exhibited the highest MCC (Matthews' correlation coefficient) values

*Note*: Data are presented as mean ± standard deviation or median (interquartile range). CAD, coronary artery disease; AMI, acute myocardial infarction; MI, myocardial infarction; Gal-3, galectin-3; AF, atrial fibrillation; NSTEMI , non-ST elevation myocardial infarction; RF, radiofrequency; CRP, C-reactive protein; NOAF, new-onset atrial fibrillation; POAF, postoperative atrial fibrillation; LVEF, left ventricular ejection fraction; CABG, coronary artery bypass grafting.

## Data Availability

The datasets used and/or analyzed during the current study are available from the corresponding author upon reasonable request.
